# Rivaroxaban for the treatment of cerebral venous thrombosis

**DOI:** 10.1186/s12883-021-02091-1

**Published:** 2021-02-15

**Authors:** Sara Esmaeili, Meysam Abolmaali, Sobhan Aarabi, Mohammad Reza Motamed, Samira Chaibakhsh, Mohammad Taghi Joghataei, Mohammad Mojtahed, Zahra Mirzaasgari

**Affiliations:** 1 Student Research Committee, Iran University of Medical Sciences, Tehran, Iran; 2 Cellular and Molecular Research Center, Iran University of Medical Sciences, Tehran, Iran; 3 School of Advanced Technologies in Medicine, Iran University of Medical Sciences, Tehran, Iran; 4 Department of Neurology, Firoozgar Hospital, Iran University of Medical Sciences, Tehran, Iran; 5 Eye Research Center, The Five Senses Institute Rassoul Akram Hospital, Iran University of Medical Sciences, Tehran, Iran

**Keywords:** Cerebral venous thrombosis, Rivaroxaban, Warfarin, Recanalization, Bleeding risk

## Abstract

**Background:**

New Oral Anticoagulants (NOACs) such as Rivaroxaban are introduced as alternatives to conventional vitamin-K antagonists in the long-term treatment of thrombotic events due to their lower bleeding risk. There is a lack of evidence on the effectiveness and safety of Rivaroxaban in Cerebral venous thrombosis (CVT). This study aims to assess the effectiveness and bleeding risk of Rivaroxaban in comparison with Warfarin for the treatment of CVT.

**Materials and methods:**

36 patients with diagnosis of CVT were included. Clinical and background information was assessed on admission and patients were followed for at least 12 months. Measured outcomes were modified Rankin Scale (mRS), evidence of recanalization on contrast-enhanced Brain MR venography (MRV) and major or minor bleeding. Patients were divided into two groups according to the type of oral anticoagulant (Rivaroxaban vs Warfarin). Groups were compared in terms of final outcomes and side effects.

**Result:**

Overall, 13 (36.11%) patients received Warfarin and 23 (63.89%) received Rivaroxaban. Optimal mRS score (0–1) was attained in 9 of 10 (90%) of patients treated with Rivaroxaban and 19 of 22 (86.36%) of patients received Warfarin. MRV showed complete or partial recanalization in 12 of 14 (85.71%) patients treated with Rivaroxaban and all patients in the Warfarin group. There was no significant difference between the two groups in terms of major and minor hemorrhage.

**Conclusion:**

Rivaroxaban holds promise for the treatment of CVT.

## Background

Cerebral venous thrombosis (CVT) is a clinical condition in which the brain’s venous drainage is impaired. This, in turn, may lead to brain ischemia or hemorrhage [1]. The incidence of CVT is 2 to 5 cases per million per year in the population [2] and it accounts for about 0.5% of strokes [3].

Diagnosis of CVT is confirmed via appropriate imaging modalities, including Brain CT scan and Magnetic Resonance Venography (MRV) [4]. Administration of heparin or Low Molecular Weight Heparin (LMWH) followed by an anticoagulant is the current approved management in CVT patients [5]. Warfarin is the most frequently prescribed medication [4]; However, it has its own limitations need for continuous monitoring, bleeding risk, and variable bioavailability due to dietary interaction [6].

Over the last decades, Novel Oral Anticoagulants (NOACs) such as Rivaroxaban might offer a convenient alternative to Warfarin in some thrombotic and thromboembolic disorders [7, 8] Some evidence shows that NOACs generally reduce the potential risks of major and fatal hemorrhages, the major adverse event of anticoagulant drugs, compared to vitamin-K antagonists [9–11]. Some reports have shown that Rivaroxaban has a therapeutic effect equivalent to Warfarin in deep vein thrombosis and pulmonary embolism [12, 13]. It has been recently presumed that it could be possible to use Rivaroxaban in cases of CVT as well; however, this concept is relatively new with insufficient evidence. Thus, its utilization in the CVT treatment is still under debate. Only a few reports have addressed this issue; they show similar clinical efficacy of factor Xa inhibitors to Warfarin for CVT treatment and reduced risk for complications [14–16]. Ongoing RCT studies comparing Rivaroxaban and Warfarin are underway (NCT03178864, and NCT04569279).

Since Rivaroxaban’s benefits may exceed that of Warfarin due to no need for continuous monitoring and no dietary interaction, this study is an attempt to address the question of whether Rivaroxaban can be a viable alternative to warfarin or not.

## Materials and methods

**Fig. 1:**
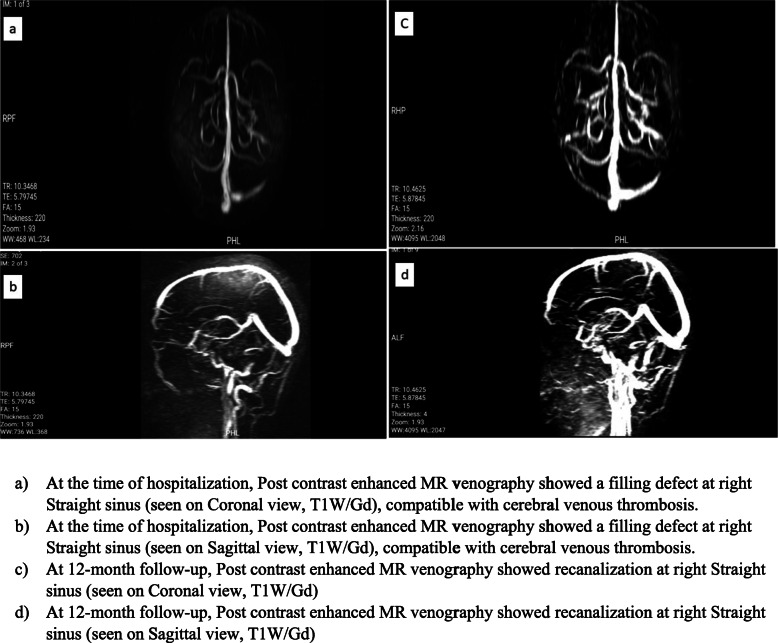
Contrats-enhanced Magnetic Resonance venography of a patient with Cerebral Venus Thrombosis (CVT). At the time of hospitalization and at 12-month follow-up. **a**. At the time of hospitalization, coronal (**a**) and sagittal (**b**) contrast-enhanced MR venography showed a filling defect in the right transverse sinus, compatible with cerebral venous thrombosis. At 12-month follow-up, coronal (**c**) and sagittal (**d**) contrast enhanced MR venography showed recanalization of right transverse sinus

In this retrospective study, we included 36 patients admitted to a central neurology hospital affiliated to Iran University of Medical Sciences, Tehran, Iran from 2017 to 2020 with cerebral venous/sinus thrombosis. The diagnosis had been confirmed by filling defects at sinuses on post-contrast enhanced Brain MR venography (MRV) (Fig. 1 a,b). Initial relevant demographic data and clinical features including predisposing factors, clinical findings, imaging characteristics, the prescribed anticoagulant drug at the time of hospitalization, and discharge were obtained from patients’ documents. Classification of patients with hemorrhagic brain lesion were done according to European Cooperative Acute Stroke Study (ECASS) [17].

During the hospitalization, all patients were treated with either Heparin or LMWH (Enoxaparin). After the initial phase, oral anticoagulants started either Warfarin or Rivaroxaban (as an off-label medication). None of the clinical symptoms, predisposing factors including hypercoagulation state, or imaging findings played a role in the type of drug (Rivaroxaban or Warfarin) prescribed by the physicians and the selection was solely made according to physician and patients’ preference. The patients' glomerular filtration rate (GFR) was calculated based on renal creatinine clearance and Rivaroxaban dose was adjusted accordingly. The initial dose of Rivaroxaban was 20 mg in patients with normal renal function and it was maintained if side effects did not occur. Warfarin was started at 5–10 mg, and further increased based on serial INR results. Dosage adjustment in patients was performed according to the European Heart Rhythm Association (EHRA) protocol in patients with renal insufficiency. Renal function was monitored regularly according to the protocol. In patients with GFR above 60 ml/min renal function was assessed every 12 months. In patients with GFR < 60 recheck interval was calculated by dividing creatinine clearance by 10 (recheck interval = CrCl/10) [18].

Patients were divided into two groups according to oral anticoagulant (Warfarin vs. Rivaroxaban). Patients’ follow-up began 12 months after the hospitalization period. They were followed up for treatment assessments and possible side effects through face-to-face evaluation by a staff neurologist. Follow-ups included clinical evaluation in terms of modified Rankin Scale (mRS), and inquiry about the occurrence of any other events of CVT during therapy. Furthermore, the occurrence of any type of bleeding was noted. In cases of bleeding, the severity and complications were assessed.MRS 0 or 1 were considered as favorable outcome [15]. 3 patients were missed to follow-up, thereby were not included in the final analysis. Similarly, the final contrast-enhanced brain MR venography was assessed for detecting recanalization. (Fig. 1 c,d) The MRV results were categorized into two groups of complete and partial recanalization. Subsequently, both groups were compared in terms of the final outcomes and side effects.

This research was conducted according to institutional and national policies. Consent forms were obtained from patients prior to using their clinical data. Patient records were obtained and included anonymously. All stages of the project were approved by the Research Ethics Committee (Code: IR.IUMS.REC.1399.436).

The results of quantitative statistics were expressed as mean and standard deviations, while qualitative data was presented by their frequency (percent). To assess the relationship between categorical variables, the Fisher exact or Chi-square test was utilized. Mann–Whitney test was used to compare quantitative variables between two groups. Statistically, *P*-value < 0.05 was considered significant. All data were analyzed by SPSS version 26 software.

## Results

A total of 36 patients were included, among which 29 were female (80.56%). The mean age was 35.69 ± 10.87. Three patients were missed to follow-up due to their unwillingness to participate in the study, thus excluded for further analysis (Table 1).

**Table 1:** Demographic and clinical background

Variables	All patients (*n* = 36)	Warfarin (*n* = 13)	Rivaroxaban (*n* = 23)	*P* value
Age	36	34 ± 11.22	36 ± 11.15	0.510
Sex	36	Male: 1 (7.69%) Female: 12 (92.31%)	Male: 6 (26.9%) Female: 17 (73.1%)	0.382
Predisposing factors				
OCP or hormone therapy	7 (19.44%)	3 (23.1%)	4 (17.4%)	0.050
Predisposing genetic hyper coagulopathy	12 (33.33%)	7 (53.8%)	5 (21.7%)	
Malignancy	4 (11.11%)	0	4 (17.39%)	
Cerebral lesion				0.708
Ischemic	25 (69.4%)	10 (76.9%)	15 (65.2%)	
Hemorrhagic	11 (36.6%)	3 (23.1%)	8 (34.8%)	
PHI	8 (81.82%)	3 (100%)	6 (75%)	
PHII	2 (18.18%)	0 (0%)	2 (25%)	

*OCP* Oral Contraceptive Pill, *PH* Parenchymal Hemorrhage (European Cooperative Acute Stroke Study classification) Quantitative statistics were shown using Mean and Standard Deviations, Mean ± (SD)

After 12 months follow-up, 9 of 10 (90%) of patients treated with Rivaroxaban and 19 of 22 (86.36%) of patients treated with Warfarin had a favorable mRS score (0 or 1). There was no statistically significant difference between the two groups in terms of the final mRS score (*P*-value> 0.05). None of the patients in either group died during the 12 months. One patient in the Rivaroxaban group experienced another CVT during the treatment period. Also, one patient in the Warfarin group experienced Deep Vein Thrombosis (DVT) during the treatment period. Concerning Imaging, MRV showed that recanalization either complete or partial, occurred in 12 of 14 (85.71%) of patients treated with Rivaroxaban and 100% of patients treated with Warfarin. The MRV in all patients of the warfarin group showed recanalization and just two patients (14.29%) in the group of Rivaroxaban had no recanalization. This difference between the two groups was not statistically significant (*P*-value> 0.05) (Table 2).

**Table 2:** Clinical findings, side effects and treatment outcomes

Clinical findings	All patients (36)	Warfarin (13)	Rivaroxaban (23)	P value
Duration of hospitalization (days)		16.77 ± 10.69	11.7 ± 7.61	0.133
Loss of consciousness	7 (19.44%)			0.382
Yes		1 (7.7%)	6 (26.1%)	
No		12 (92.3%)	17 (73.9%)	
Outcomes of treatment				
Minor bleeding	5 (15.63%)	2/10 (20%)	3/22 (13.63)	0.708
Major bleeding	0	0	0	
mRS	32^a^	10	22	> 0.999
mRS (0 or 1)	28 (87.5%)	9 (90%)	19 (86.36%)	
mRS 2	4 (12.5%)	1 (10%)	3 (13.63%)	
MRV (Recanalization)	19^a^	5	14	> 0.999
Recanalized	17 (93.75%)	5 (100%)	12 (85.71%)	
Complete	14 (84.37%)	4 (80%)	10 (83.33%)	
Partial	3 (9.38%)	1 (20%)	2 (16.67%)	
Not recanalized	2 (6.25%)	0	2 (14.29%)	

*mRS* modified ranking scale, *MRV* magnetic resonance venography. Quantitative statistics were shown using Mean and Standard Deviations, Mean ± (SD)

^a^ The number of the statistical population in the follow-up (mRS and MRV) was lower than the initial population due to not answering the phone call or unwillingness to participate in follow-up

Three patients treated with Rivaroxaban experienced minor bleeding and 2 cases experienced the same in the other group. The difference between the two groups was not statistically significant (*P*-value> 0.05). There was no report of any major bleedings as side effects of Warfarin or Rivaroxaban. Bleedings were all cured without further complications after dose adjustment.

## Discussion

This study aimed to compare the benefits and risks of Rivaroxaban with Warfarin in patients with cerebral venous thrombosis (CVT).

In this study, anticoagulant therapy was maintained by either Rivaroxaban, or dose-adjusted Warfarin for 12 months. As stated, none of the clinical symptoms, predisposing factors inducing hypercoagulation state or imaging findings played a role in the type of drug (Rivaroxaban or Warfarin) prescribed by the physicians, and the drug option was solely selected based on physician and patients’ preference. The initial dose of Rivaroxaban was 20 mg and it was maintained unless bleeding occuredoccured [19].

Both drugs were rarely associated with major bleeding events, there have been no new intracranial hemorrhages nor any expansion of initial hemorrhagic lesions. No significant bleeding risk was observed with Rivaroxaban compared to Warfarin; this result was similar previuos the studies on Rivaroxaban used for CVT or other thrombotic diseases such as Deep Vein Thrombosis or Pulmonary Thromboembolism [15, 16, 20–22].

Patient outcomes were measured by the mRS score and thrombosis recanalization by MR venography. Despite the small sample size, the groups did not differ significantly in terms of final clinical outcome and evidence of recanalization in MRV. Specifically, 90% of patients treated with Rivaroxaban had a favorable mRS score of 0 or 1 which seems to be, albeit insignificantly, higher than the warfarin group. Only one case in the Rivaroxaban group experienced new venous/sinus thrombosis. Inquiries revealed that this patient changed the dose of Rivaroxaban to 10 mg, and did not continue the advised 20 mg dosage. No death was reported in either group during 12 months follow-up.

Moreover, 85.7% of patients in the Rivaroxaban group showed complete recanalization in MRV after 12 months while, recanalization occurred in 100% of the Warfarin group. This difference was not significant between the two groups. Previous studies, either case reports or small population studies on CVT, have shown high efficacy of factor Xa-inhibitor drugs, especially in the case of Rivaroxaban. Resulting in high rates of recanalization and optimal mRS score (0–1) [15, 16, 20, 23].

The present research findings on side effects and clinical outcomes are consistent with those of previous studies on Rivaroxaban for the treatment of thrombotic disorders other than CVT (12). An important consideration in comparing different studies are predisposing factors including sex, age, and history of thrombophilia or hypercoagulable state. Further, the duration of follow-up may play a determining role in clinical outcomes. For example, recanalization rates in our study were superior to those reported by Geisbusch et al. and Lurkin et al. [15] and this can be due to our follow-up time, 12 months, which is longer than the mentioned studies (8 and 6 months respectively) [15, 24] . Also, Lurkin et al. [24], used different types of NOAC drugs other than Rivaroxaban, including Apixaban or Dabigatran.

### Limitations

Since the present work is a retrospective study with small sample size, the findings should be deduced with caution. Furthermore, prescribing Rivaroxaban or Warfarin according to preference of related physician limits our study. Future prospective studies with a larger study group should be performed.

## Conclusion

The efficacy of Rivaroxaban in CVT might not be inferior to vitamin K antagonists. The risk of bleeding in patients treated with Rivaroxaban, as a major concern of anticoagulant therapy, seems not to be superior to that of Warfarin.

## Data Availability

The datasets generated and/or analyzed during the current study are not publicly available due to ethic consideration and policy of our center but are available from the corresponding author on reasonable request.
